# An interpretable multiparametric radiomics model of basal ganglia to predict dementia conversion in Parkinson’s disease

**DOI:** 10.1038/s41531-023-00566-1

**Published:** 2023-08-30

**Authors:** Chae Jung Park, Jihwan Eom, Ki Sung Park, Yae Won Park, Seok Jong Chung, Yun Joong Kim, Sung Soo Ahn, Jinna Kim, Phil Hyu Lee, Young Ho Sohn, Seung-Koo Lee

**Affiliations:** 1 Department of Radiology, Yongin Severance Hospital, Yonsei University Health System, Yongin-si, Gyeonggi-do South Korea; 2 Department of Computer Science, Yonsei University, Seoul, South Korea; 3 Department of Mechanical Engineering, Pohang University of Science and Technology, Pohang, Republic of Korea; 4 Department of Radiology and Research Institute of Radiological Science and Center for Clinical Imaging Data Science, Yonsei University College of Medicine, Seoul, South Korea; 5 Department of Neurology, Yongin Severance Hospital, Yonsei University Health System, Yongin-si, Gyeonggi-do South Korea; 6 Department of Neurology, Yonsei University College of Medicine, Seoul, South Korea; 7 YONSEI BEYOND LAB, Yongin-si, Gyeonggi-do South Korea

**Keywords:** Parkinson's disease, Parkinson's disease, Prognostic markers

## Abstract

Cognitive impairment in Parkinson’s disease (PD) severely affects patients’ prognosis, and early detection of patients at high risk of dementia conversion is important for establishing treatment strategies. We aimed to investigate whether multiparametric MRI radiomics from basal ganglia can improve the prediction of dementia development in PD when integrated with clinical profiles. In this retrospective study, 262 patients with newly diagnosed PD (June 2008–July 2017, follow-up >5 years) were included. MRI radiomic features (*n* = 1284) were extracted from bilateral caudate and putamen. Two models were developed to predict dementia development: (1) a clinical model—age, disease duration, and cognitive composite scores, and (2) a combined clinical and radiomics model. The area under the receiver operating characteristic curve (AUC) were calculated for each model. The models’ interpretabilities were studied. Among total 262 PD patients (mean age, 68 years ± 8 [standard deviation]; 134 men), 51 (30.4%), and 24 (25.5%) patients developed dementia within 5 years of PD diagnosis in the training (*n* = 168) and test sets (*n* = 94), respectively. The combined model achieved superior predictive performance compared to the clinical model in training (AUCs 0.928 vs. 0.894, *P* = 0.284) and test set (AUCs 0.889 vs. 0.722, *P* = 0.016). The cognitive composite scores of the frontal/executive function domain contributed most to predicting dementia. Radiomics derived from the caudate were also highly associated with cognitive decline. Multiparametric MRI radiomics may have an incremental prognostic value when integrated with clinical profiles to predict future cognitive decline in PD.

## Introduction

Cognitive impairment is a common non-motor symptom of Parkinson’s disease (PD), and approximately 80% of patients develop dementia within 20 years of diagnosis^1^. Dementia significantly affects the morbidity and mortality in PD, and early detection of patients at high risk of dementia conversion is important for proper implementation of therapeutic and supportive strategies^2^. Although the neurobiology underlying the cognitive decline in PD remains unclear, nigrostriatal degeneration is the core pathologic feature of PD^3^, and basal ganglia are likely to play a major role in the development of cognitive decline. Ample evidence suggests that dopamine deficiency in frontostriatal circuits is associated with early executive dysfunction in patients with PD^4^. In particular, the caudate has been proposed as a strong candidate associated with cognitive function in PD^5^. A recent study showed that a preferential dopamine loss in the anterior putamen was associated with a greater risk of developing PD with dementia (PDD)^6^. Further, several MRI studies have reported that structural^7,8^ and functional changes^9^ in the basal ganglia are associated with cognitive decline in PD.

Radiomics is an advanced technology extracting high-dimensional quantitative imaging features, such as intensity distributions, textural heterogeneity, and shape descriptors^10^. Radiomics aims to discover meaningful “hidden” information within radiological images, which is visually inaccessible to clinicians. The strength of radiomics is that it can reveal intralesional heterogeneity by quantification of texture information through mathematical extraction of the spatial distribution of signal intensities and pixel interrelationship^11^. In this study, we hypothesized that a combination of clinical information and radiomic features derived from MRI can help to accurately identify patients at a high risk of PDD. We investigated whether a multiparametric radiomics model of the basal ganglia (putamen and caudate) can improve the PDD prediction in patients with PD when integrated with clinical profiles.

## Results

### Clinical characteristics of patients with PD

The baseline clinical characteristics of the 262 patients with PD in the training set (*n* = 168) and test set (*n* = 94) are summarized in Table 1. In all, 51 (30.4%) and 24 (25.5%) patients developed PDD within 5 years of PD diagnosis in the training and test sets, respectively. In both training and test sets, patients who developed PDD within a defined time window were older, predominantly male, and had higher UPDRS-III scores compared with the characteristics of patients who did not develop PDD. The patients who developed PDD showed lower K-MMSE scores (*P* < 0.001) and lower composite scores for the visual memory/visuospatial function (*P* = 0.004 and 0.003, respectively), verbal memory function (*P* = 0.002 and 0.051, respectively), and frontal/executive function domains (*P* < 0.001) compared to those who did not develop PDD.

**Table 1:** Baseline clinical characteristics of the study participants.

Clinical variables	Training set (*N* = 168)			Test set (*N* = 94)			*P* value^b^
	No PDD	PDD	*P* value^a^	No PDD	PDD	*P* value^a^	
	*n* = 117	*n* = 51		*n* = 70	*n* = 24		
Age (years)	66.2 ± 6.9	71.3 ± 7.5	<0.001	66.6 ± 8.4	76.2 ± 6.1	<0.001	0.212
Onset age (years)	64.9 ± 7.3	69.5 ± 7.9	<0.001	64.8 ± 8.4	74.7 ± 5.8	<0.001	0.295
Female, no. (%)	62 (53.0%)	16 (31.4%)	0.016	42 (60.0%)	8 (33.3%)	0.043	0.357
Education (years)	8.8 ± 4.5	9.9 ± 4.8	0.146	10.2 ± 3.9	7.3 ± 4.8	0.004	0.608
Time from symptom onset to diagnosis (months)	15.9 ± 14.0	22.0 ± 18.3	0.020	20.7 ± 19.2	18.0 ± 13.8	0.526	0.286
UPDRS-III	21.2 ± 10.2	25.1 ± 11.6	0.030	21.7 ± 8.7	25.6 ± 7.1	0.047	0.794
Cognitive performance							
K-MMSE (/30)	27.3 ± 2.2	25.8 ± 2.7	<0.001	27.2 ± 1.9	23.6 ± 3.1	<0.001	0.096
Visual memory/visuospatial^c^	0.06 ± 0.90	−0.43 ± 1.16	0.004	0.11 ± 0.81	−0.47 ± 0.77	0.003	0.695
Verbal memory^c^	−0.01 ± 1.05	−0.57 ± 1.00	0.002	0.19 ± 0.93	−0.24 ± 0.94	0.051	0.044
Frontal/executive^c^	0.17 ± 1.03	−0.85 ± 0.96	<0.001	0.26 ± 0.91	−0.85 ± 0.88	<0.001	0.406
Attention/working memory/language^c^	0.00 ± 1.17	−0.01 ± 1.32	0.968	−0.16 ± 0.84	−0.57 ± 0.75	0.034	0.065

*PD* Parkinson’s disease, *PDD* Parkinson’s disease with dementia, *UPDRS-III* Unified PD Rating Scale Part III, *K-MMSE* the Korean version of the Mini-Mental State Examination.

Values are expressed as mean ± standard deviation or number (percentage).

^a^Comparisons between patients with PD who progressed to dementia within 5 years after the diagnosis of PD and those who did not develop dementia within 5 years.

^b^Comparisons between the training and test sets.

^c^The composite scores of each cognitive function domain were calculated according to the formula described in the previous work^14^.

The follow-up period was significantly longer in the training set compared to the test set (median 8.0 vs. 5.6 years, *P* = 0.012), which was expected as the training and test sets were allocated temporally. There were no significant differences between the training and test sets with regard to the age, sex, educational attainment, duration of PD, and the UPDRS-III scores. Cognitive performances were not significantly different between the training and test set, except for the verbal memory function.

### Selected features and model performances

The multivariable regression analysis revealed that among clinical features, age and the composite scores of visuospatial/visual memory, verbal memory, and frontal/executive function domains had significant associations with dementia development, except the disease duration, without multi-collinearity. The detailed results are presented in Supplementary Table 1.

The performances of models for the prediction of PDD development in the training and test sets are provided in Table 2. In the training set, the clinical model showed an AUC, accuracy, sensitivity, and specificity of 0.894 (95% confidence interval [CI], 0.845–0.943), 82.4%, 74.5%, and 85.5%, respectively. In the test set, the AUC, accuracy, sensitivity, and specificity were 0.722 (95% CI, 0.606–0.838), 73.4%, 58.3%, and 78.6%, respectively.

**Table 2:** The performances of two models for prediction of PDD conversion in the training and test sets.

	AUC (95% CI)	Accuracy (%)	Sensitivity (%)	Specificity (%)	*P* value	NRI
Training set						
Clinical model	0.89 (0.85–0.94)	82.4	74.5	85.5		
Clinical + Radiomics model	0.93 (0.89–0.97)	84.5	78.4	87.2	0.284	0.119
Test set						
Clinical model	0.72 (0.61–0.84)	73.4	58.3	78.6		
Clinical + Radiomics model	0.89 (0.82–0.96)	79.8	75.0	81.4	0.016	0.207

*PDD* Parkinson’s disease with dementia, *AUC* area under the curve, *CI* confidence interval, *NRI* net reclassification index.

**Fig. 1:**
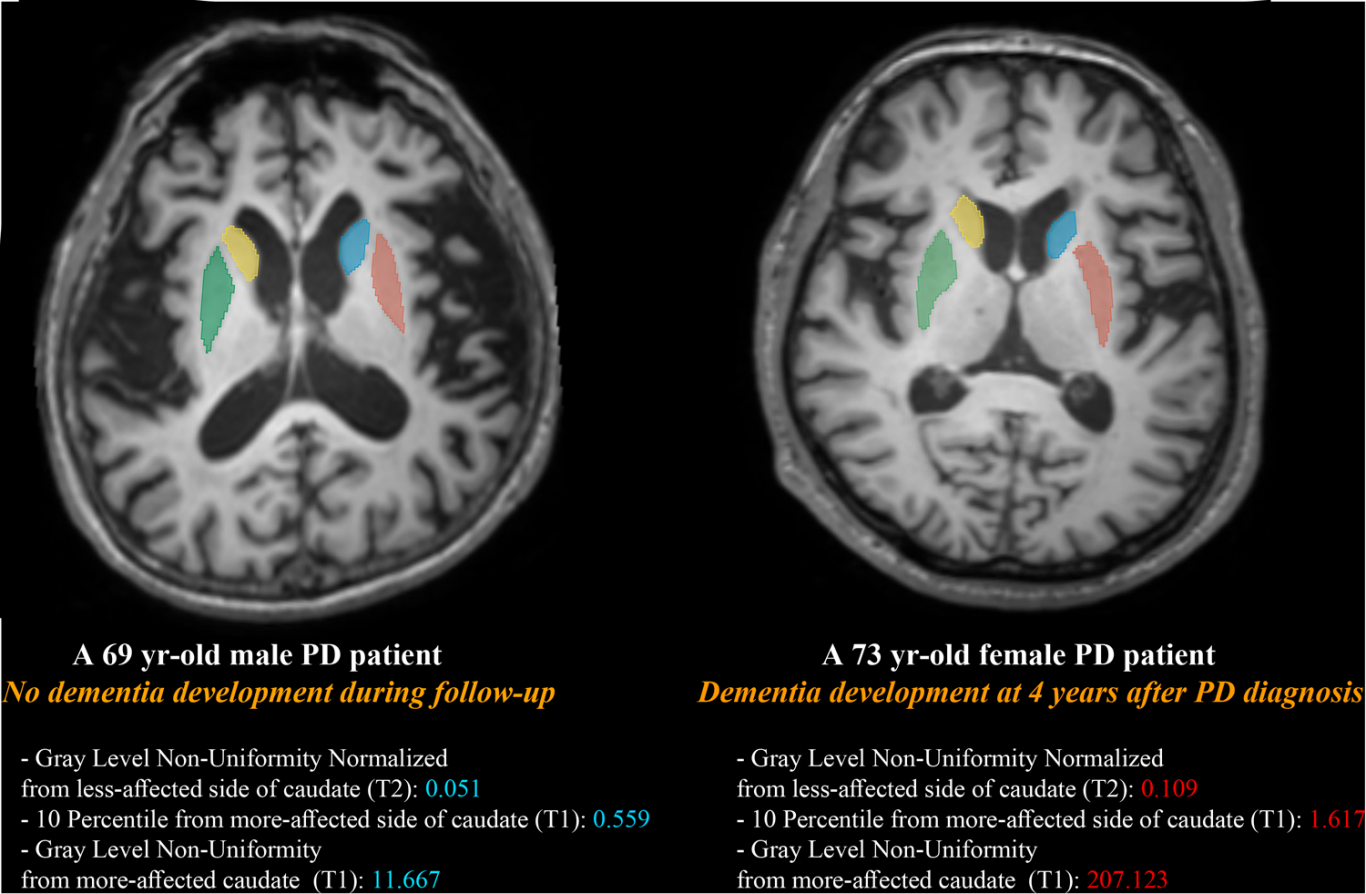
The representative figures from two patients with and without dementia development with their radiomic feature values. Region of interests were drawn on both sides of caudate and putamen. A 69-year-old male who did not develop dementia during the follow-up period showed overall lower scores of selected three radiomic features compared to those from a 73-year-old female who develop dementia.

In the combined clinical and radiomics model, a total of eight features were selected: five clinical features (age, disease duration, composite scores of visuospatial/visual memory, verbal memory, and frontal/executive function domains) and three radiomic features (Gray-Level Non-Uniformity Normalized from the less-affected side of the caudate [GLRLM feature from T2], 10 Percentile from the more-affected side of the caudate [first-order feature from T1], and Gray Level Non-Uniformity from the more-affected caudate [GLDM feature from T1]). The results of Pearson correlation analysis between the selected radiomic features and clinical features are presented in Supplementary Table 2. The representative figures from two patients with and without dementia development with their values of selected radiomic features are provided in the Fig. 1. In the training set, the AUC, accuracy, sensitivity, and specificity were 0.928 (95% CI, 0.890–0.967), 84.5%, 78.4%, and 87.2%, respectively. In the test set, the AUC, accuracy, sensitivity, and specificity were 0.889 (95% CI, 0.820–0.959), 79.8%, 75.0%, and 81.4%, respectively.

**Fig. 2:**
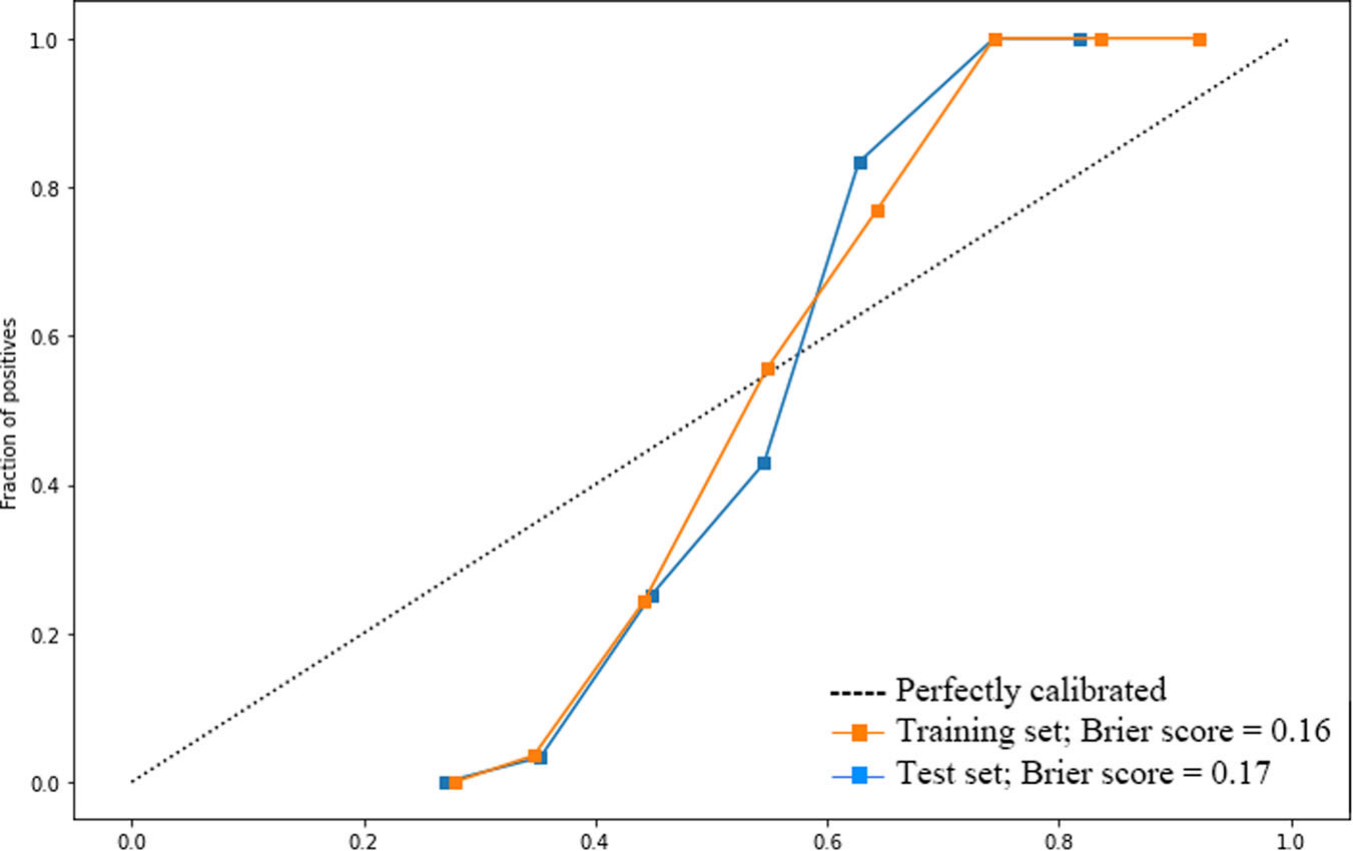
Calibration curves and Brier scores of the combined model (clinical + radiomic features) in both training and test sets. The Brier score was 0.16 and 0.17 in the training and test set, respectively.

Calibration curves of the combined models were obtained (Fig. 2), demonstrating relatively good consistency between the estimated and actual probability of dementia conversion in both training and test sets. We also calculated the goodness of a predicted probability score with Brier score, which is between 0.0 and 1.0, where a model with perfect accuracy has a score of 0.0 and the worst has a score of 1.0. The Brier score was 0.16 and 0.17 in the training and test set, respectively.

### Comparison of model performances

**Fig. 3:**
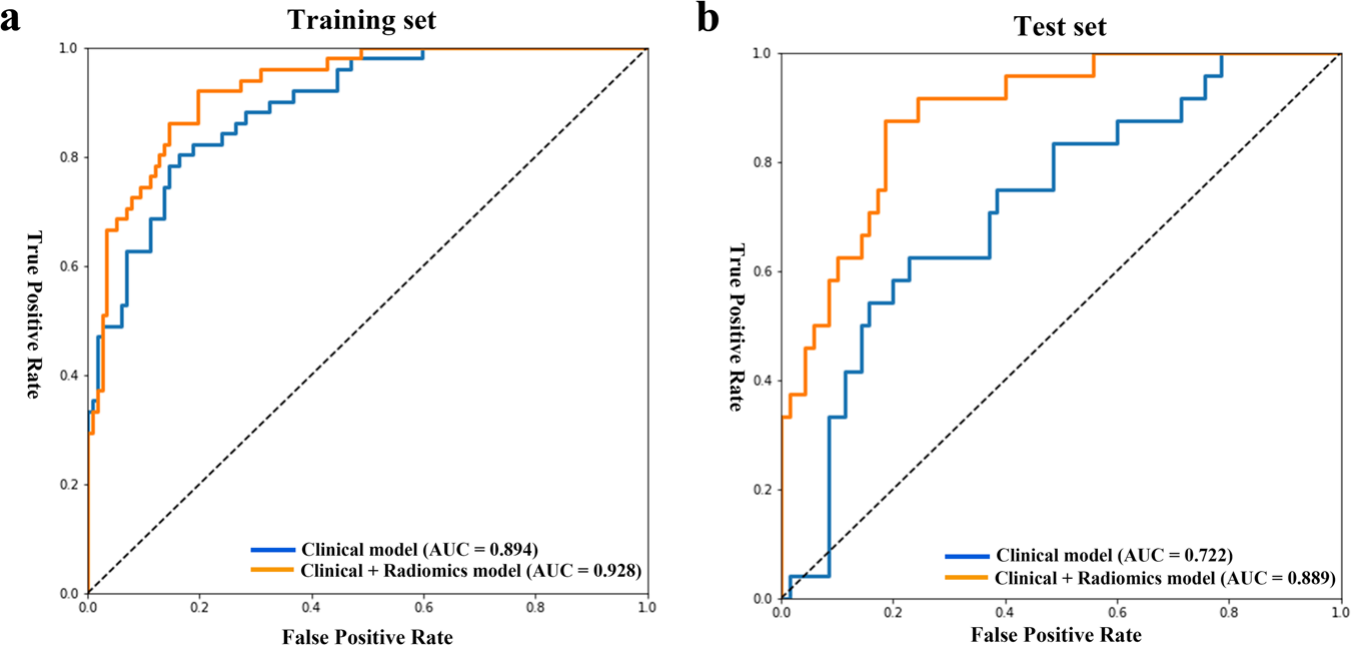
Receiver operating characteristics curves of the models in the training and test sets. **a** In the training set, the combined clinical and radiomics model tended to show superior performance compared to that of the model with only clinical features (AUC: 0.928 vs. 0.894, *P* = 0.284, NRI = 0.119). **b** In the test set, the performance of the combined clinical and radiomics model was superior to that of the clinical model (AUC: 0.889 vs. 0.722, *P* = 0.016, NRI = 0.207). AUC area under the curve, NRI net reclassification index.

In the training set, the combined clinical and radiomics model tended to show superior performance compared to that of the model with only clinical features (AUC: 0.928 vs. 0.894, *P* = 0.284, NRI = 0.119). In the test set, the performance of the combined clinical and radiomics model was superior to that of the clinical model (AUC: 0.889 vs. 0.722, *P* = 0.016, NRI = 0.207) (Table 2 and Fig. 3).

### Model interpretability with SHAP

**Fig. 4:**
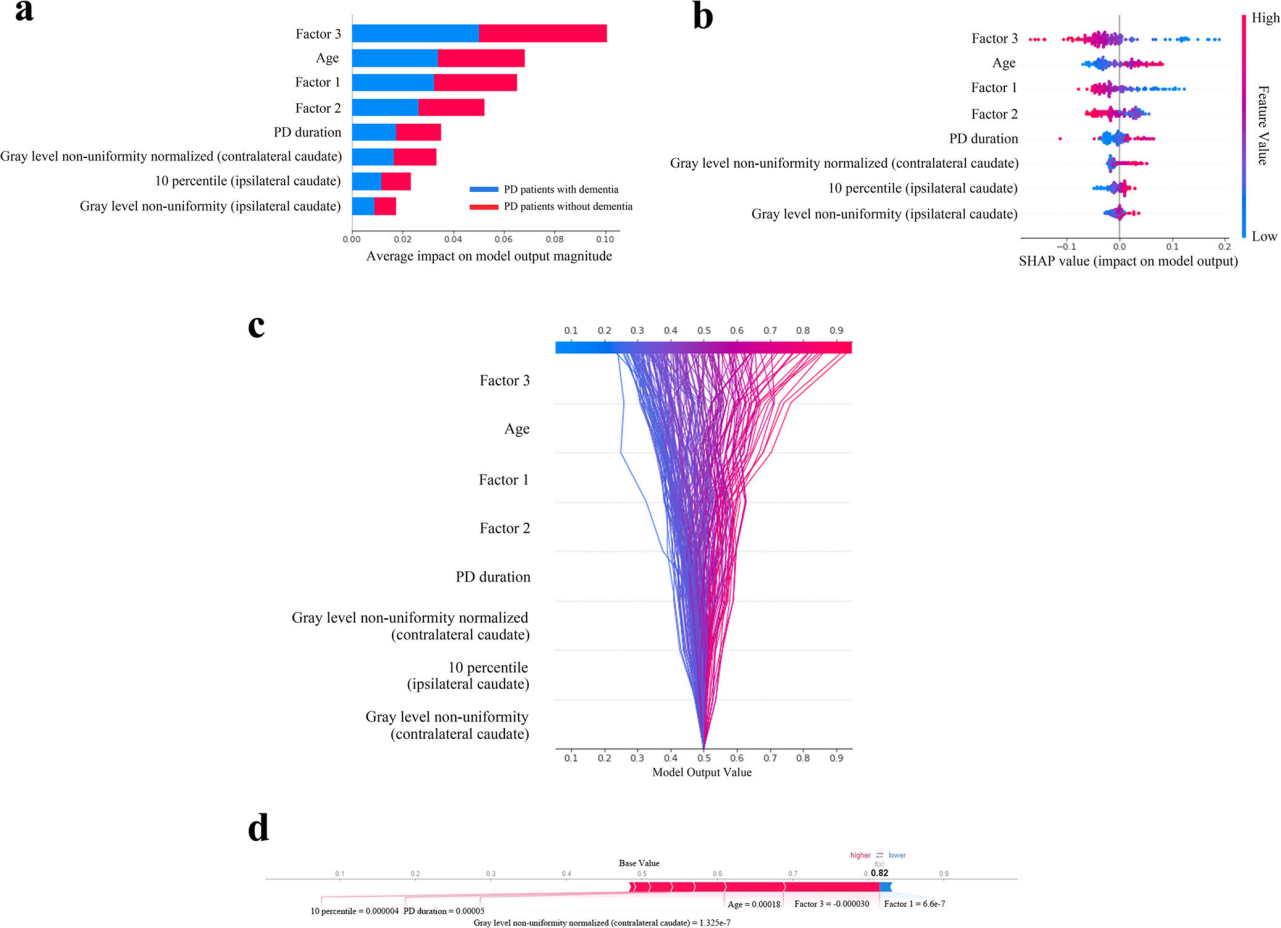
Model interpretability of the combined clinical and radiomics model for the prediction of dementia conversion with SHapley Additive exPlanations (SHAP) in the training set. **a** Variance importance plot listing the most significant variables. Features with greater importance for the prediction of dementia conversion are positioned in the upper portion, and the features are presented in descending order. **b** Summary plot of feature impact on the decision of the model showing positive and negative relationships of the predictors with the target variable. A positive SHAP value indicates an increase in the probability of dementia conversion. **c** Decision plot showing how the model predicts dementia conversion. Starting at the bottom of the plot, the prediction line shows how the SHAP values accumulate from the base value to arrive at the model’s final score at the top of the plot, demonstrating how each feature contributes to the overall prediction. **d** Force plot of a representative patient who developed dementia during the follow-up period. Red arrows represent features that drive the prediction value higher, while blue arrows represent features that drive the prediction value lower. The size of each arrow represents the magnitude of the effect of the corresponding feature. Note that factor 3 and age largely push the model prediction score higher. Factor 1 visual memory/visuospatial function, Factor 2 verbal memory function, Factor 3 frontal/executive function.

The SHAP values for each selected feature in the combined clinical and radiomics model were calculated, and the relevant plots are shown in Fig. 4. For each prediction, a positive SHAP value indicates an increase in the risk of developing PDD. The plots show that composite scores of the frontal/executive function domain were the most important risk factors, followed by age and composite scores of the visuospatial/visual memory and verbal memory function. Regarding the radiomic features, Gray Level Non-Uniformity Normalized from the less-affected side of the caudate [T2] was the highest contributing factor in predicting PDD.

## Discussion

In this study, we investigated whether the MRI radiomic features of the basal ganglia can improve the prediction of the development of dementia in patients with PD when integrated with a machine-learning classifier. As a result, several key clinical and radiomics features with significant association with PDD conversion were identified. We also found that the combined model of radiomics and clinical features achieved a superior performance for predicting PDD conversion compared to the clinical model (AUC 0.889 vs. 0.722 in the test set).

Cognitive impairment is commonly observed in patients with PD even at the early stages and can severely affect the quality of life and function, which necessitates identification of predictors of future cognitive decline in PD^12^. Several predictors have been proposed as markers for ongoing cognitive decline in PD, including age, genetic variation in APOE and MAPT, gait disturbance, motor assessments, non-motor symptoms, electroencephalogram analysis results, cognitive profiles, as well as several plasma biomarkers (e.g., α-synuclein/Aβ40, MIA, CRP, and albumin)^13–16^. In addition, several neuroimaging studies have shown that structural and functional integrity measured by MRI data can be a useful marker for early dementia conversion in patients with PD^8,17–20^. Our previous works also demonstrated that cortical thinning in the frontal areas and disrupted white matter connectivity in frontal and posterior cortical regions were associated with early dementia conversion in patients with PD^18,20^. However, so far, inconsistent results have been reported for both cortical thickness analyses and diffusion tensor imaging analyses, and there are no validated neuroimaging biomarkers yet. Radiomics, which enables mining of high-dimensional quantitative imaging features, has been frequently addressed in medical fields, specifically in the field of neurodegenerative diseases including PD. Numerous previous studies pointed out that radiomics can predict the diagnosis of PD^21,22^, motor handicap^23^, identify PD subtypes^24^, and predict PD progression assessed by Hoehn-Yahr Scale^25^. Therefore, based on this potential of radiomics, we hypothesized that radiomic features derived from classical MRI parameters may provide complementary information to predict PDD development. A few recent publications attempted to predict cognitive decline in PD with radiomics and suggested its prognostic role^26,27^, with applying radiomics to either T1^27^ or quantitative susceptibility mapping^26^. In our study, multiparametric radiomic features from T1, T2, and FLAIR images were extracted for a relatively larger sample size, allowing for a more comprehensive analysis. Further, radiomic features were integrated with well-known clinical features to identify the added prognostic value of radiomics, which was also validated in an independent test set. Our results showed that multiparametric MRI radiomics, considered together with the clinical profile, has the potential to predict the development of dementia in patients with PD.

Among the selected features from the combined clinical and radiomics model, the Gray Level Non-Uniformity Normalized feature of GLRLM from the less-affected side of caudate, significantly contributed to the prediction of PDD conversion. The Gray Level Non-Uniformity feature measures the similarity of gray-level intensity values in an image, such that a higher value correlates with lesser similarity and greater heterogeneity^28^. Previous studies have reported that patients with PDD tend to exhibit iron deposition in the caudate^29^ and have a greater burden of cerebral microbleeds compared with patients without cognitive decline^30^. In addition, a higher severity in scoring of enlarged perivascular spaces in basal ganglia was associated with cognitive decline in PD^8^. Therefore, the frequently observed MRI findings in PD with cognitive decline may be attributed to the heterogeneity in the caudate, which might be captured by extracted radiomic features. Interestingly, the corresponding feature extracted from the less-affected side of the caudate rather than the more-affected side contributed the most to prediction of PDD conversion. Although the exact mechanism is unclear, much evidence has shown that the less-affected striatum also demonstrates considerable degree of degeneration, reduced endogenous dopamine, reduced dopamine uptake, and reduced fiber integrity, as assessed using PET, MR spectroscopy, and diffusion tensor imaging^31^. Further, the less-affected striatum appears to provide compensatory support to maintain the dopaminergic activity in the more-affected striatum, through crossed nigrostriatal pathways and alterations in subthalamic activity^32^. Therefore, the radiomic feature from the less-affected side of the caudate may provide clinically relevant information to predict PDD conversion.

In terms of the clinical variables, the frontal/executive function was the single most significant factor for the prediction of dementia. Several previous studies have attempted to identify neuropsychological predictors for PDD, yielding heterogeneous results. All cognitive domains, including the frontal/executive, visuospatial, memory, and language functions, have been associated with early PDD conversion^2^. A large community-based cohort study from the United Kingdom^33^, proposed that posterior cortical dysfunction, but not frontostriatal deficits, is a predictor for early dementia conversion in PD. Meanwhile, our previous works supported that the frontal/executive dysfunction would make a greater contribution to the development of PDD than dysfunction in other cognitive domains^14,18,20^. These discrepant findings likely reflect the marked clinical heterogeneity of PD^14^. The results of the present study are consistent with those of our previous works^14,18,20^, which highlighted the contribution of frontal/executive dysfunction to the early development of PDD, even when the radiomic features from the basal ganglia are additionally included as predictors. Although the exact mechanism remains to be elucidated, impairment of the frontal/executive function or frontal-subcortical pathways may further affect other cognitive domains through disruption of the reciprocal cortico-cortical connections or important nodes of information integration^34^.

In our study, we attempted to predict whether the patients develop dementia or not and performed classification analysis for the prediction of binary outcomes, rather survival analysis which predicts time to dementia development. Unlike determining the survival in cancer patients, the estimation of the time of dementia conversion in PD could be inaccurate, even though we made a great effort to determine whether patients progressed to dementia at every visit. Given that a considerable number of patients with PD eventually develop dementia and each patient enrolled in this study had a different follow-up period, we employed a 5-year time window for the determination of dementia development. The time from the diagnosis of PD to dementia conversion was treated as a categorical variable (i.e., whether a patient developed dementia within 5 years of PD diagnosis) in the model, rather than a continuous variable for the Cox proportional hazards model in the survival analysis. Indeed, in studies of patients with PD, binary classification tasks are frequently performed to predict dementia conversion^20,35,36^. Further, rather using a conventional statistical method such as binary logistic regression analysis, we applied machine-learning techniques in our study. Regression analysis is designed for relatively small datasets, and is not suitable when the number of features or variables exceeds the number of observations (i.e., high-dimensional datasets)^37^. Regression analysis can also be applied in the radiomics studies if appropriate feature selection methods can be preceded, however, we chose machine-learning techniques for the analysis as it is a more flexible alternative for analyzing high-dimensional, right-censored, and heterogeneous data^37^. Machine-learning techniques inherently handle high-dimensional data and have been adapted to handle censored data, therefore, can give more accurate results than traditional statistical methods when modeling high-dimensional data.

For the comparison of model performances, we used the two statistical methods: DeLong’s method and NRI. NRI was proposed either as an alternative or a supplement to C-index, as C-index has been criticized as being relatively insensitive to changes in absolute risk estimates and therefore having little power to detect modest but potentially meaningful differences between risk models^38,39^. Together with DeLong’s method, NRI is one of the widely used statistics for the assessment of the two models’ relative ability to discriminate between events and nonevents by quantifying the agreement between “upward” and “downward” risk reclassifications and event status^40,41^. In the training set, adding radiomics to the clinical model did not significantly enhance the model performance when the performances were compared with DeLong’s method. It may be attributed to the fact that the pure clinical model performed well enough with high AUC, comparable to that of the combined model, and the difference in AUCs was subtle. However, NRI proved the superiority of the combined clinical and radiomics model. In the test set, it was noteworthy that the combined model maintained a superior performance therefore. the AUCs of the clinical and the combined clinical and radiomics model exhibited a significant difference when assessed by both DeLong’s method and NRI. We believe that our study proved the added prognostic value of radiomics with adequate statistics and validation.

There are several limitations in our study. First, it was a single-center, retrospective study. Further studies with a larger dataset and external validation are needed to evaluate the generalizability of the models. Second, we used an automatic pipeline for brain segmentation (i.e., volBrain), which simply divided the basal ganglia into the putamen and caudate. More detailed segmentation of the striatum is needed to elucidate the association between other striatal sub-regions (e.g., anterior putamen and ventral striatum) and the risk for PDD conversion^5,6^.

In conclusion, we developed a model based on clinical and radiomic features to predict dementia conversion within 5 years of PD diagnosis. Its performance was superior to that of the model based only on clinical profiles. These findings suggest that clinical profiles and multiparametric MRI radiomics integrated with machine-learning classifiers may help predict future cognitive decline in patients with PD.

## Methods

### Participants

We retrospectively reviewed the Yonsei Parkinson Center database for medical records of 293 consecutive patients with newly diagnosed PD who first visited the outpatient clinic at Severance Hospital between June 2008 and July 2017. All the patients had been followed up for more than 3 years. PD was diagnosed according to the clinical diagnostic criteria of the United Kingdom PD Society Brain Bank^42^. All patients underwent brain MRI and detailed neuropsychological tests at the initial assessment. All subjects underwent a standardized neuropsychological battery called the Seoul Neuropsychological Screening Battery (SNSB) at initial assessment^43^. The SNSB covers five cognitive domains: attention and working memory (forward/backward digit span task and letter cancellation); language and related functions (the Korean version of the Boston Naming Test [K-BNT], calculation, and praxis); visuospatial function (the Rey Complex Figure Test [RCFT] copy), verbal and visual memory (immediate recall/delayed recall/recognition test using the Seoul Verbal Learning Test [SVLT] for verbal memory; immediate recall/delayed recall/recognition test using the RCFT for visual memory); and frontal/executive function (contrasting program and go/no-go test, the Controlled Oral Word Association Test [COWAT], and the Stroop test). To reduce the redundancy of neuropsychological subtests and the possibility of overrepresenting a single cognitive function domain, we first conducted a factor analysis based on age- and education-specific z-scores of 14 scorable subtests of the SNSB (forward digit span task, backward digit span task, K-BNT, RCFT copy, immediate recall, delayed recall, and recognition items using the SVLT and RCFT, COWAT for animal, COWAT for supermarket, COWAT for phonemic fluency, and the Stroop color reading test) to yield four cognitive function domains (visual memory/visuospatial [factor 1], verbal memory [factor 2], frontal/executive [factor 3], and attention/working memory/language [factor 4]) in patients with PD^14^. The calculating formula are as follows:

Visual memory/visuospatial function = 0.422 × RCFT (immediate recall) + 0.417 × RCFT (delayed recall) + 0.259 × RCFT copy + 0.179 × RCFT (recognition) − 0.033 × SVLT (delayed recall) − 0.098 × SVLT (recognition) − 0.056 × SVLT (immediate recall) − 0.096 × COWAT-semantic fluency [supermarket] − 0.048 × COWAT-semantic fluency [animal] − 0.026 × COWAT-phonemic fluency + 0.076 × Color Stroop test - 0.148 × Forward digit span − 0.072 × Backward digit span + 0.034 × K-BNT.

Verbal memory function = −0.016 × RCFT (immediate recall) − 0.014 × RCFT (delayed recall) − 0.138 × RCFT copy − 0.025 × RCFT (recognition) + 0.436 × SVLT (delayed recall) + 0.437 × SVLT (recognition) + 0.378 × SVLT (immediate recall) − 0.030 × COWAT-semantic fluency [supermarket] − 0.020 × COWAT-semantic fluency [animal] − 0.073 × COWAT-phonemic fluency − 0.090 × Color Stroop test − 0.043 × Forward digit span − 0.061 × Backward digit span − 0.001 × K-BNT.

Frontal/executive function = −0.054 × RCFT (immediate recall) − 0.034 × RCFT (delayed recall) + 0.058 × RCFT copy − 0.149 × RCFT (recognition) − 0.060 × SVLT (delayed recall) − 0.126 × SVLT (recognition) + 0.059 × SVLT (immediate recall) + 0.405 × COWAT-semantic fluency [supermarket] + 0.373 × COWAT-semantic fluency [animal] + 0.315 × COWAT-phonemic fluency + 0.305 × Color Stroop test − 0.111 × Forward digit span + 0.022 × Backward digit span + 0.017 × K-BNT.

Attention/working memory/language function = −0.156 × RCFT (immediate recall) − 0.163 × RCFT (delayed recall) + 0.026 × RCFT copy + 0.227 × RCFT (recognition) − 0.069 × SVLT (delayed recall) + 0.039 × SVLT (recognition) − 0.098 × SVLT (immediate recall) − 0.107 × COWAT-semantic fluency [supermarket] − 0.071 × COWAT-semantic fluency [animal] + 0.081 × COWAT-phonemic fluency − 0.055 × Color Stroop test + 0.593 × Forward digit span + 0.449 × Backward digit span + 0.233 × K-BNT.

Parkinsonian motor symptoms were assessed using the Unified Parkinson’s Disease Rating Scale Part III (UPDRS-III), and the sum of the scores of the UPDRS-III items was calculated for each side of the body to identify the more-affected side.

**Fig. 5:**
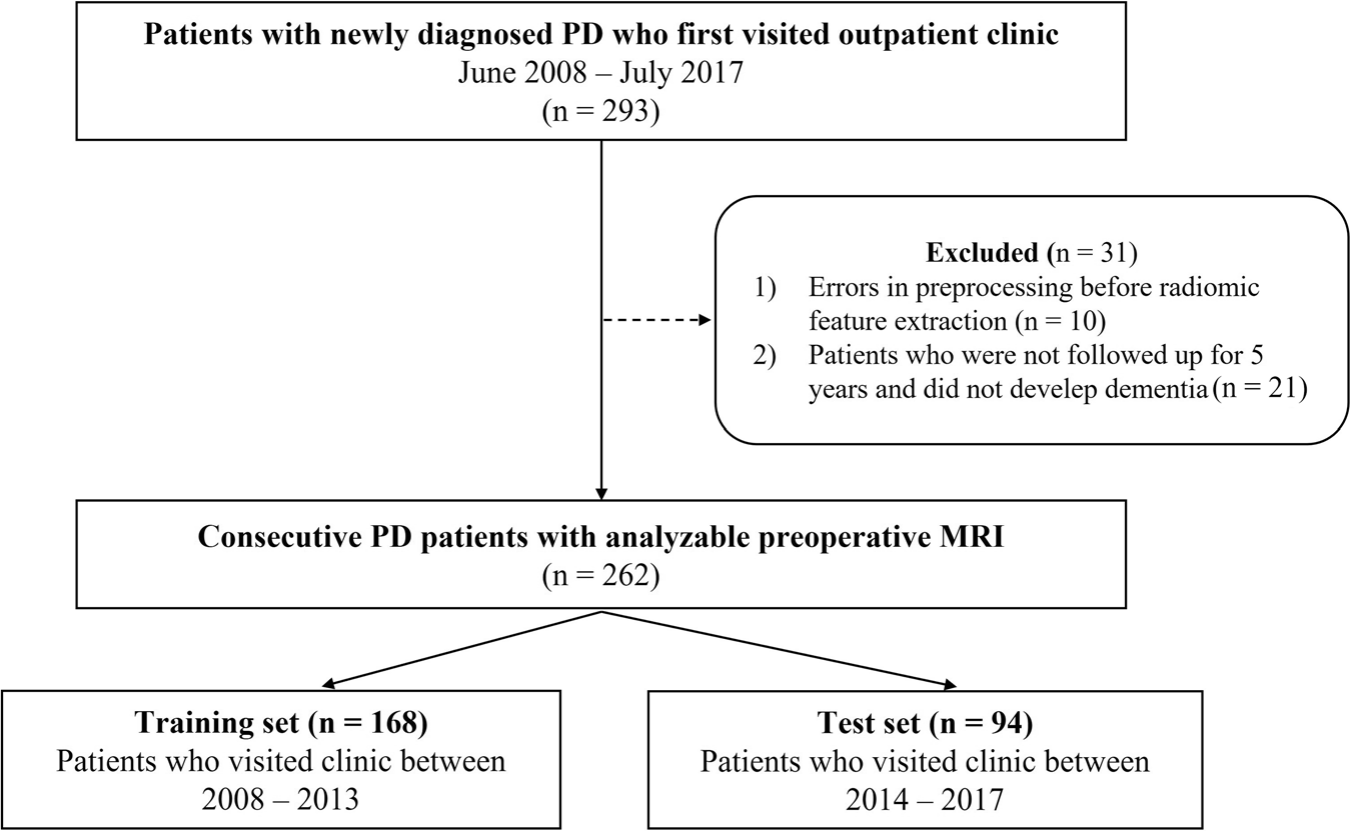
Flowchart of patient enrollment.

Among 293 patients, 21 (7.2%) patients were not followed up for the full 5 years and did not develop dementia until they were lost to follow-up. In addition, 10 patients were excluded from the study due to errors in the MRI dicom files, which resulted in failures of radiomic feature extraction. Thus, a total of 262 patients with PD were included in the final study population. Patients who visited the clinic between 2008 and 2013 were allocated to the training set (*n* = 168), and the patients who visited the clinic between 2014 and 2017 were allocated to the test set (*n* = 94) to perform external temporal validation (Fig. 5).

### Standard protocol approvals, registration, and patient consents

This study was approved by the Yonsei University Severance Hospital institutional review board (4-2022-0650), and the need for informed consent was waived due to the retrospective nature of the study.

### Assessment of dementia conversion

During the follow-up period, patients were diagnosed with PDD if they fulfilled the clinical criteria for probable PDD based on the Movement Disorder Society Task Force guidelines^14,44^. After diagnosis of PD, patients visited the outpatient clinic at 3-month intervals, and at every visit, they or their caregivers were asked questions regarding their daily functioning. Additionally, all patients underwent serial cognitive assessment using the Korean version of the Mini-Mental State Examination (K-MMSE) and Clock Drawing Test with a one-year interval (Level I tests)^44^. In case of definite cognitive decline or evidence of impairments in daily life due to cognitive changes (Level I^45^), most patients underwent the SNSB to identify the pattern of cognitive deficits and diagnose PDD at Level II^44,46^.

Since a considerable number of patients with PD eventually develop PDD^1,47^, a definitive time window is needed to determine whether the patient is at high risk of developing PDD^18^. A 5-year time window was employed based on previous studies^14,20^. Whether the patients developed PDD during the 5-year of follow-up period was investigated. Among the 262 patients with newly diagnosed PD, 75 patients had progressed to PDD within 5 years after the diagnosis of PD.

### MRI protocols

All scans were acquired with a 3T scanner (Achieva; Philips Healthcare, Best, the Netherlands, or Ingenia CX; Philips Healthcare, Best, the Netherlands) with a 32-channel head coil. Head motion was minimized with restraining foam pads provided by the manufacturer. The MRI imaging protocol included T2-weighted images (repetition time [TR]/echo time [TE], 2800–3000/80–100 ms; field of view [FOV], 230–240 mm; section thickness, 5 mm; slice gap, 7 mm; matrix, 256 × 256), FLAIR (TR/TE, 9000–10,000/110–125 ms; FOV, 240 mm; section thickness, 5 mm; slice gap, 7 mm; matrix, 256 × 256), and noncontrast 3D T1-weighted images (TR/TE, 6.9/3.2 ms; FOV, 230–240 mm; section thickness, 1.2 mm; matrix, 256 × 256).

### Image preprocessing and radiomic feature extraction

**Fig. 6:**
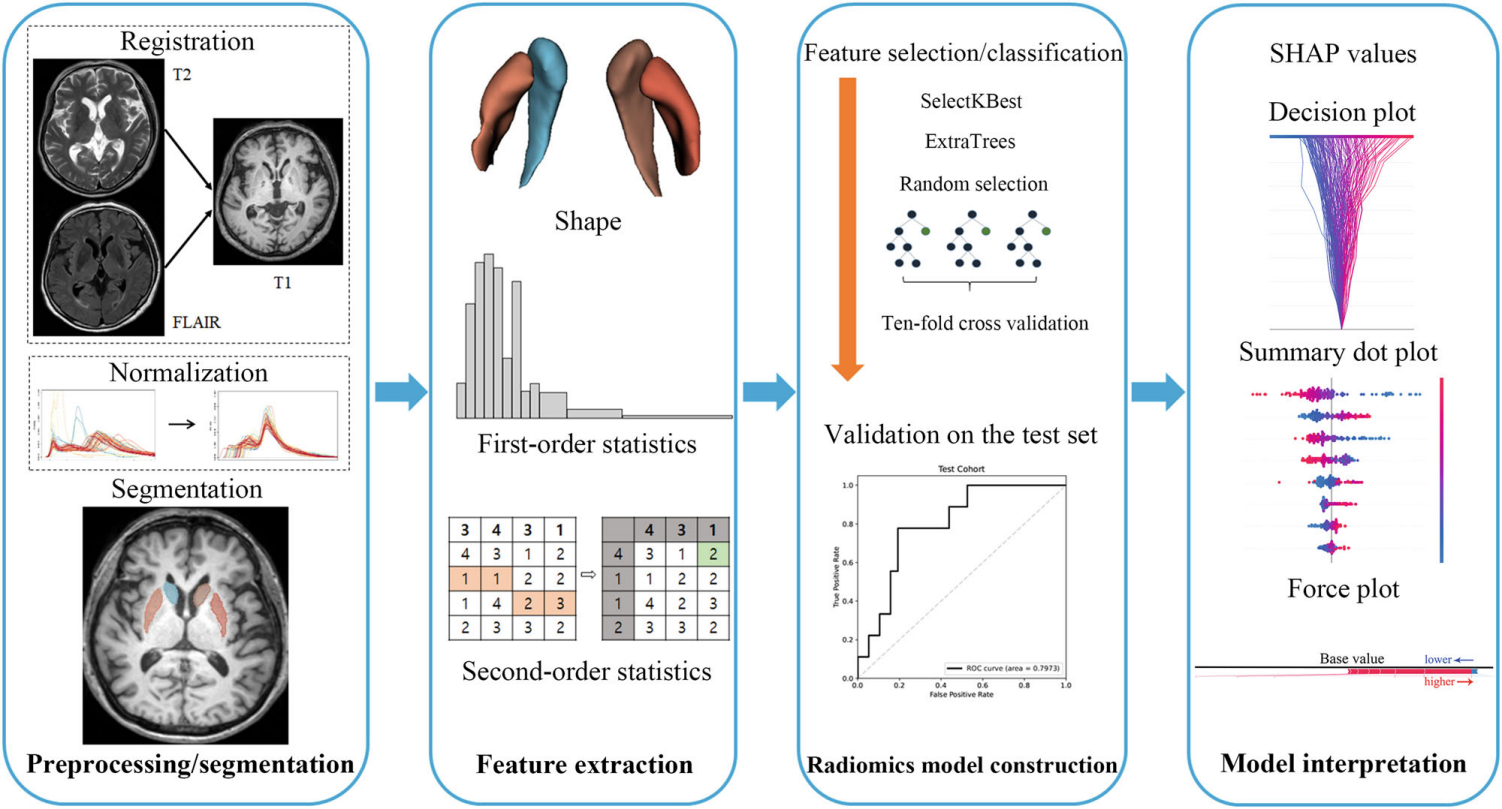
Workflow of image preprocessing, radiomics feature extraction, and machine learning. (1) Preprocessing and segmentation: For the radiomic feature extraction, registration of T2 and FLAIR to T1 images and normalization of signal intensities was performed. The regions of interest were put on the bilateral putamen and caudate. (2) Feature extraction: Three different categories of radiomic features—shape feature, first-order features, and second-order features were obtained. (3) Radiomics model construction: SelectKBest feature selection method combined with ExtraTrees classifier were used to develop two predictive models—clinical and combined (clinical + radiomics) model. The models were developed in the training set, then validated in the test set. (4) Model interpretation: We performed SHAP analysis to understand the contributing role of each selected radiomic feature and obtained decision plot, summary dot plot, and force plot.

The detailed processes of image preprocessing and radiomic feature extraction are described in Fig. 6. Automated mask extraction of the basal ganglia, namely putamen and caudate, was performed using volBrain (https://volbrain.upv.es/)^48^, which is a robust automatic pipeline for brain segmentation with high accuracy^49^. Preprocessing of the images was performed to standardize the data analysis across patients. After removing unwanted low-frequency intensity non-uniformity by applying the N4 bias correction algorithm^50^, normalization of signal intensity was performed via z-score. All images were resampled to 1-mm isovoxels. T2 and FLAIR images were co-registered with T1 images by affine transformation with normalized mutual information as a cost function.

After image preprocessing, radiomic feature extraction from bilateral caudate and putamen was performed using PyRadiomics (version 2.0)^51^, which conformed to the Image Biomarker Standardization Initiative^52^. Based on the more-affected side of each patient (either right or left), radiomic features from the more-affected caudate or putamen were distinguished from those of the less-affected caudate or putamen. The radiomic features included 14 shape features, 18 first-order features, and 75 second-order features [such as gray-level co-occurrence matrix (*n* = 24), gray-level run-length matrix (GLRLM, *n* = 16), gray-level size zone matrix (*n* = 16), gray-level dependence matrix (GLDM, *n* = 14), and neighboring gray tone difference matrix (*n* = 5)]. A total of 1284 (107 features × 2 sub-regions of the basal ganglia (caudate and putamen) × more-affected/less-affected side × 3 sequences) radiomic features were extracted.

### Machine learning and model construction

Feature selection and machine-learning process were performed using Python 3 with the Scikit-Learn library module (version 0.21.2). Because the number of radiomic features was greater than the number of cases, the SelectKBest function in the Scikit-Learn module was used for feature selection according to the k highest scores^53^. Then, selected radiomic features were integrated with the ExtraTrees classifier to build a predictive model with ten-fold cross-validation. In the ten-fold cross-validation, the training set is split into 10 folds. A fold is used in each iteration once as testing data, while the remaining folds are used as training data^54^. The process is repetitive until all dataset is evaluated, and the cross-validation results in the average performance of the models.

Two types of models were trained as follows: (1) a clinical model—age, disease duration, cognitive composite scores of visual memory/visuospatial, verbal memory, and frontal/executive function domains, and (2) a combined model based on radiomics and clinical features. The clinical features to predict PDD conversion within 5 years of PD diagnosis were selected based on the Cox regression analysis results of our previous study^14^. The two models were developed from the training set and were validated in the test set. The multivariable regression analysis was performed in 262 PD patients to examine whether each clinical feature had independent and significant associations with the development of dementia. Pearson correlation analysis was performed between the selected radiomic features and clinical features to evaluate whether they have a significant correlation. The area under the receiver operating characteristic curve (AUC), accuracy, sensitivity, and specificity were obtained. Additionally, the calibration curves of the combined model (clinical and radiomic features) were plotted in both training and test sets to examine the models’ accuracy, together with Brier score. Calibration refers to the agreement between observed outcomes and predictions^55^. A calibration plot is the primary graphical method for evaluating calibration performance. A graphical assessment of calibration is possible with predictions on the *x* axis, and the outcome on the *y* axis. Perfect predictions should be on the 45° line. A slope close to 1 and an intercept close to 0 (i.e., the 45° line of the plot) indicates good calibration^56^. For linear regression, the calibration plot is a simple scatter plot. For binary outcomes, the plot contains only 0 and 1 values for the *y* axis^57^. Smoothing techniques can be used to estimate the observed probabilities of the outcome (p(*y* = 1)) in relation to the predicted probabilities.

The Brier score is not a measure of either discrimination performance or calibration performance alone, but a measure of overall performance, which incorporates both the discrimination and calibration aspects of a model that predicts binary outcomes^58^. Therefore, it is desirable to present both the Brier score and the calibration plot.

The Brier score is calculated as follows:

$${\rm{Brier}}\,{\rm{score}}=\frac{1}{n}\mathop{\sum }\limits_{i=1}^{n}{({p}_{i}-{o}_{i})}^{2}$$

where *n* is the number of subjects, *p*_i_ is the probability of event predicted by the model for the *i*th subject, and *o*i is the observed outcome in the *i*th subject (i.e., 1 for event or 0 for non-event)^57^. Therefore, a score closer to 0 indicates a better predictive performance.

The AUCs of those two models were compared by DeLong’s method^59^ and the net reclassification index (NRI)^60^. A NRI value greater than zero indicates superior performance of a new model over an old model. Multiple comparisons were corrected using a false-discovery rate approach, and a false-discovery rate-corrected *P* value < 0.05 was considered statistically significant. All statistical analysis was performed using statistical software R (version 4.0.1; R Foundation for Statistical Computing, Vienna, Austria).

### Model interpretability with Shapley Additive exPlanations (SHAP)

SHAP was used to interpret and evaluate the significance of each radiomic feature from the radiomics model^61^. SHAP, originating from game theory, assesses the contribution of each variable of the model to its output^61,62^. The output of each possible combination of other variables is collected. SHAP analysis enables the quantification of continuous and categorical variables in the texture features only and the combined models. Features listed higher on the left vertical axis indicate a stronger influence on the overall model outcome. Feature values are color-coded: red data points indicate higher values, and blue data points indicate lower values^63^. In addition, this allows the quantification of the impact of each variable on the prediction, not only on a global level (on the overall population) but also locally (on a subset or one patient)^64^. Thus, Shapley values for each variable are additive, which makes the contribution of each variable convertible to a share of the output classification probability. This provides an intuitive visualization for clinicians using this model. SHAP measured the contribution of each feature of the model to the increase or decrease in the probability of PDD development within a 5-years’ time window.

### Supplementary information

Supplementary Information

## Data Availability

All data and codes used for this study is available from the corresponding author on request.
