# Supplementary material for: An interpretable multiparametric radiomics model of basal ganglia to predict dementia conversion in Parkinson’s disease
Source: NPJ Parkinsons Dis. 2023 Aug 30;9:127. doi: 10.1038/s41531-023-00566-1 (PMC10468504; doi:10.1038/s41531-023-00566-1)
Supplement: Supplementary file 1 — Supplementary Information [file 41531_2023_566_MOESM1_ESM.pdf]

Supplementary Information

Supplementary Table 1. Logistic regression analysis for dementia within 5 years of PD diagnosis

	Beta (SE)	p-value	VIF
Overall series			
Age	0.083 (0.023)	<0.001	1.127
Disease duration	0.008 (0.010)	0.423	1.008
Visual memory/visuospatial	-0.662 (0.179)	<0.001	1.063
Verbal memory	-0.585 (0.166)	<0.001	1.024
Frontal/executive	-1.111 (0.200)	<0.001	1.113

VIF = variance inflation factor

Supplementary Table 2. The results of correlation analysis among features

1) Correlation between the selected radiomic features and clinical features

		10 Percentile from the more-affected side of the caudate	Gray Level Non-Uniformity from the more-affected caudate	Gray Level Non-Uniformity Normalized from the less-affected side of the caudate
age	correlation coefficient	-.095	-.462	.066
	P value	.124	**.000**	.290
PD duration	correlation coefficient	-.069	.023	-.006
	P value	.265	.705	.921
factor1	correlation coefficient	-.026	.058	-.067
	P value	.680	.347	.281
factor2	correlation coefficient	.164	.060	.147
	P value	.008	.335	.017
factor3	correlation coefficient	.083	.266	.118
	P value	.182	**.000**	.056

As there were 15 pairs of comparisons, the Bonferroni correction was applied, and the p-value for determining significance was adjusted to 0.05/15 = 0.0033. Therefore, the only statistically meaningful relations were found between the two pairs:

1) Age and Gray Level Non-Uniformity from the more-affected caudate

2) Factor 3 and Gray Level Non-Uniformity from the more-affected caudate

2) Variance inflation factor (VIF) for each feature included in the combined model

Variables	VIF
age	1.374
PD duration	1.018
factor1	1.056
factor2	1.065
factor3	1.149
10 Percentile from the more-affected side of the caudate	1.110
Gray Level Non-Uniformity from the more-affected caudate	1.350
Gray Level Non-Uniformity Normalized from the less-affected side of the caudate	1.092

As all the VIFs were under 5, we concluded that there were no significant correlations that require attention.

3) Pearson correlation analysis between the selected radiomic features and the volume of region of interests

		Contralateral caudate volume	Ipsilateral caudate volume	Contralateral putamen volume	Ipsilateral putamen volume
10 Percentile from the more-affected side of the caudate	Correlation coefficient	.065	.034	.012	.072
	P value	.294	.579	.848	.247
Gray Level Non-Uniformity from the more-affected caudate	Correlation coefficient	-.079	-.047	-.014	.051
	P value	.200	.450	.815	.408
Gray Level Non-Uniformity Normalized from the less-affected side of the caudate	Correlation coefficient	.021	-.010	-.041	-.044
	P value	.734	.875	.511	.474

As the radiomic feature was extracted from ipsilateral/contralateral and caudate/putamen, respectively, the ROI volume was divided into four categories, as described in the Table above. As there were 12 pairs of comparisons, the Bonferroni correction was applied, and the p-value for determining significance was adjusted to 0.05/12 = 0.0042. There were no statistically significant association between the ROI volumes and selected radiomic feature values.
